# Towards the assessment of financial distress among cancer patients: a conceptual model of the financial effects of a tumour disease

**DOI:** 10.1007/s00520-026-10395-6

**Published:** 2026-02-12

**Authors:** Sophie Pauge, Andrea Züger, Luise Richter, Viktoria Mathies, Bastian Surmann, Thomas Ernst, Natalja Menold, Wolfgang Greiner, Eva C. Winkler, Katja Mehlis

**Affiliations:** 1 Department for Health Economics and Health Care Management, School of Public Health, Bielefeld, University, Bielefeld, Germany; 2 National Center for Tumor Diseases (NCT), NCT Heidelberg, a partnership between DKFZ and Heidelberg University Hospital, Germany, Heidelberg University, Medical Faculty Heidelberg, Department of Medical Oncology, Section Translational Medical Ethics, Heidelberg, Germany; 3 Institute of the History, Theory and Ethics of Medicine, Justus-Liebig-University, Gießen, Germany; 4 Methods in Empirical Social Research, Faculty of Arts, Humanities and Social Science, Institute of Sociology, Dresden University of Technology, Dresden, Germany; 5 Department of Hematology/Oncology, Jena University Hospital, Jena, Germany; 6 Institute for Medical and Data Ethics, Heidelberg University, Faculty of Medicine, Heidelberg University Hospital, Heidelberg, Germany

**Keywords:** Financial toxicity, Cancer, Qualitative research, Patient-reported outcome, Patients, Social services, Financial distress, Coping strategies, Conceptual model

## Abstract

**Purpose:**

A conceptualisation of subjective financial distress as a consequence of a cancer diagnosis and treatment is still missing due to a lack of a comprehensive model accounting for all relevant dimensions of financial effects of cancer experienced by patients. Our goal was to derive a model for the German healthcare system to shed light on the complex process of financial effects of cancer.

**Methods:**

The model was developed through systematic literature review and qualitative studies, including interviews with 18 cancer patients and a focus group with 4 social services representatives. The iterative process of model development was accompanied by an ongoing exchange in the interdisciplinary research team.

**Results:**

The developed model of financial effects of cancer experienced by patients consists of three dimensions: (1) *actual and anticipated financial disadvantages*, (2) *behavioural* *and cognitive coping strategies*, and (3) *subjective financial distress* conceptualised as negative effects in different aspects of daily living: *employment*, *living situation*, *family*, *social participation*, *health promoting lifestyle*, *additional personally preferred treatments*, *navigating the health system*, and a further area so-called* unspecific.*

**Conclusion:**

Subjective financial distress is driven by different financial effects of cancer experienced by patients and is perceived as negative in various aspects of daily living. While the identified categories of daily living can be observed in countries with universal healthcare coverage, the content and degree of each subcategory depend on country-specific characteristics. The introduced model can be used to inform the development of a new patient-reported outcome measure (PROM).

**Trial registration number:**

NCT05319925 (registration date, 2022-06-01)

## Introduction

Subjective financial distress is a common consequence of a cancer diagnosis and treatment which can affect patients’ health outcomes, such as a deterioration in their health-related quality of life or the development of depression, anxiety [1], and even increased mortality [2]. Subjective financial distress is often discussed within the multidimensional concept “financial toxicity” that also includes objective financial burden [3–6]. The latter can be caused by direct medical costs (e.g. for medications, therapies, or medical aids), direct non-medical costs (e.g. transportation, child care, or food costs), and indirect costs (e.g. productivity losses due to inability to work or premature death). Currently, subjective financial distress is often conceptualised by three domains comprising *material conditions* which refer to the impact on individuals’ financial spending and their financial resources; *psychosocial responses* to increased cancer-related expenses; and finally, *behavioural* *coping* with the financial burden [5]. However, the interplay between objective financial burden and subjective financial distress as well as the conceptualisation of subjective financial distress itself remains unclear. Recent research suggests that the relationship between subjective financial distress and objective financial burdens is more intricate than previously believed [7–10]. While initial understanding posited subjective financial distress as a consequence of objective financial burdens, the latest studies reveal a more complex interaction between the two factors [7, 11].

The phenomenon of objective financial burden and subjective financial distress was first discussed within the context of the US healthcare system. As a result, the definitions and instruments to measure financial effects have been primarily developed based on the US-specific characteristics. In 2017, the first cancer-specific survey instrument “COST” (comprehensive score for financial toxicity) was validated in the USA [4, 12]. Most of the 11 items focussed on the consequences of additional medical expenses and access to treatment options due to personal financial constraints, highlighting the impact of direct (non-)medical costs on subjective financial distress. The US system, however, does not provide universal healthcare coverage, which hinders equality and equity for access to healthcare[13]. In line with the concept of universal healthcare coverage, countries with publicly funded healthcare systems are in general characterised by a greater level of social security systems to mitigate financial effects, adding complexity to the concept of subjective financial distress. Therefore, the transferability of the COST instrument and other developed frameworks to countries with universal healthcare coverage is limited.

Initial studies have shown that the financial situation of cancer patients in universal healthcare systems such as Germany with a high level of social security schemes deteriorates due to a combination of direct and indirect costs. A first study on changes in the financial situation of patients in Germany reports high income losses and/or additional expenditure that are associated with a poorer quality of life and higher distress [14]. Similarly, Buettner et al. (2019) [15] and Schneider et al. (2020) [16] have demonstrated that German cancer patients face relatively high out-of-pocket costs during the course of their illness.

However, most of the currently available studies used non-validated questionnaires in small populations. As a systematic review pointed out, the quantitative results of previous studies on the financial burden of cancer patients in universal healthcare systems are difficult to compare due to the heterogeneity of the constructs’ definition and methods measuring financial effects of cancer experienced by patients [5]. While the burden of (in-)direct costs in Germany and other countries with universal healthcare coverage could be quantified in some populations and a transferability to other cancer entities can be assumed, the degree and extent of subjective financial distress and its relation to the objective financial burden remain open. To better understand the phenomenon of subjective financial distress, to understand the interplay with the objective financial burden, and to capture the extent of financial effects in German cancer patients, a comprehensive model is needed to measure financial effects precisely as a patient-reported outcome measure (PROM) in the long term. 

Therefore, the purpose of this study is to develop a conceptual model of financial effects among German cancer patients to inform the development and validation of an instrument that measures patient-reported financial effects.

## Material and methods

This study is part of the interdisciplinary project *Financial Effects of a* *Tumor* *Disease* (FIAT) and was registered under NCT05319925 on clinicaltrials.gov. The study protocol has been published [17]. In this paper, we report on the development of a conceptual model for the financial effects of cancer experienced by patients by conducting a systematic literature review and qualitative studies with patients and social services. An overview of all work packages can be found in the supplementary material.

### Systematic literature review

A systematic literature review was performed to map subjective financial distress as a PROM, where a total of 46 out of 4321 identified studies were included [18]. Methods and results of risk factors for subjective financial distress have been published in Pauge et al. [18]. In brief, the literature search wa conducted in PubMed, PsycINFO, and CINAHL up to December 2020. Studies from high-income countries with universal healthcare systems examining patients’ perception of their financial effects due to a cancer diagnosis were eligible. To analyse the construct of subjective financial distress, definitions of subjective financial distress were extracted and, following Witte et al. [5, 14]. The construct was categorised into three domains as (I) material conditions, (II) psychosocial responses, and (III) coping behaviours [18].

### Patient perspective: semi-structured interviews

Participants, totaling 18, were recruited from outpatient units in southern Germany and inpatient units in central Germany. In the outpatient setting, a study nurse informed and invited potential participants, while in inpatient care, a research associate handled recruitment. Selection criteria included employment status (self-employed, not self-employed, unemployed), insurance type (statutory, private), age, gender, and marital status, employing a theoretical sampling approach. Inclusion criteria comprised any cancer type, a minimum of 2 months of cancer-related therapy, and an ECOG (Eastern Cooperative Oncology Group) Status of less than 3. The sample was nearly balanced in gender (10 males, 8 females) and covered various cancer types. Most participants were employed, with others being self-employed, unemployed, pensioners, or students. Sources of income included salary, sick pay, unemployment benefits, pensions, and no income. The majority were married and had public health insurance. Further details on methods and demographic characteristics can be found elsewhere[19].

The interview guideline was developed based on the main research interests and relevant literature [5, 20]. The interviews were conducted between May 2021 and December 2021 by A.Z. and V.M. The interviews were analysed using qualitative content analysis by combining a deductive and inductive approach as suggested by Kuckartz [21]. Initially, pre-established codes were developed based on the research questions and existing models of financial distress. The transcripts were then analysed deductively using three codes: (1) *objective financial* *disadvantages*, (2) *coping strategies*, and (3) *subjective financial distress*.

### Social services perspective: focus group

The focus group participants were recruited through the social service of the National Center for Tumor Diseases (NCT) Heidelberg and through the mailing list of the German Association for Social Work in Health Care (Deutsche Vereinigung für Soziale Arbeit im Gesundheitswesen e.V.) based on predefined eligibility criteria to assure professional engagement with cancer patients with relevant topic on a daily business. The participants (*n* = 4) were female, full-time social service workers. Half of them worked in outpatient cancer counselling centres, while the others were employed in social services at acute care hospitals. The social services were located in three different regions in Germany. All participants worked exclusively with cancer patients without restrictions regarding cancer types treated at their site. The social workers were at least 5 years within their profession.

An interview guideline was developed in advance based on results of systematic reviews on financial distress and a survey of social services [22]. The focus group, chaired by S.P. and B.S., took place in October 2021. Transcripts were analysed using qualitative content analysis following Kuckartz in the same manner as applied in the analysis of the semi-structured interviews [21]. Transcripts were initially coded with four categories of (1) *status quo of subjective financial distress in Germany*, (2) *social services counselling in practice*, (3) *requirements* *of a new PROM* (4) *applicability of PROM* *in practice*.

### Development of the conceptual model

Based on the findings, a conceptual model for assessing subjective financial distress was developed. Stressors determined through the systematic review were used to differentiate the construct of financial distress from its risk factors. New topics and domains as mentioned by the patients and representatives of social services were inductively identified, and their contribution to the construct of financial distress was determined to integrate it into the model. The conceptual model was developed in ongoing and iterative discussions within the interdisciplinary research team from the fields of oncology/medical ethics, health economics, and methods in empirical social research.

## Results

During the iterative process of the model development, three dimensions of financial effects were identified that partially influence each other: (1) *objective financial disadvantages*, (2) *coping strategies*, and (3) *subjective financial distress*. The results presented below describe the individual dimensions as they resulted from the overall qualitative content analysis of the different working tasks (Table 1).

**Table 1:** Overview of the process to develop a conceptual model of financial effects in Germany (Table 1)

Step	Task	Aim	Method
1	**Literature review**	To analyse the construct of subjective financial distress and the risk factors associated with experiencing financial distress	Systematic literature review in databases of PubMed, PsycINFO and CINAHL up to December 2020. Qualitative synthesis of dimensions of measured subjective financial distress and risk factors for experiencing financial distress
2	**Qualitative interviews with patients**	To understand dimensions of financial effects of cancer for the affected patients in Germany	Semi-structured interviews with cancer patients (*n *= 18) in two cancer centres in Germany. Transcripts analysed by qualitative content analysis
3	**Focus group with social services representatives**	To determine further dimensions and its risk factors of financial effects from the perspective of social services representatives	Focus group with representatives of social services (*n* = 4) from outpatient cancer counselling and acute hospitals in Germany. Transcript analysed by qualitative content analysis
4	**Definition of construct and model**	To define the construct of financial effects and the conceptual model	Qualitative content analysis of all results from previous tasks. Expert-group-discussions within the interdisciplinary research team with expertise in oncology/medical ethics, health economics and empirical social research

### Objective financial disadvantages

Objective financial disadvantages persist as *indirect costs*, *direct medical costs*, and *direct non-medical costs.* Direct medical costs refer to costs that occur in the healthcare setting for the delivery of healthcare while direct non-medical costs arise additionally for accessing healthcare services outside the healthcare system (e.g. transportation costs, childcare).Table 2 provides an overview of the direct and indirect costs and the corresponding sources from which the results originate (qualitative interviews with patients, focus group with social service representatives, systematic literature review).

**Table 2:** Objective financial disadvantages experienced by cancer patients

Category	Subcategory	Source		
		Patients	Social services	Literature review
	**Reduced financial resources:**			
**Indirect costs (financial losses)**	(Household) Income	x	x	[14, 23–31]
	Compensation payments		x	[23, 25, 28, 32]
	Savings	x		[25, 27, 30, 32–34]
**Direct medical costs**	**(Co)payments for:**			
	Hospital stay	x		[31]
	Prescription drugs, remedies and aids	x	x	[24, 31, 35–37]
	Over the counter drugs	x	x	
	Therapies, e.g. physiotherapy	x		
	Medical aids	x		
	Hair replacements	x		
	Special nutrition	x		[24, 26, 28]
**Direct non-medical costs**	**(Co)payments for:**			
	Higher costs of living (spending more time at home)	x		[24, 29, 31, 38]
	Living costs during hospital and rehabilitation stay	x		[24, 35]
	Transportation to healthcare institutions	x	x	[24, 28, 37]
	Complementary therapies	x	x	[24]
	Home modifications			[29, 31]
	Homecare		x	[26]
	Childcare			[26, 31]

All direct (non-)medical costs refer to additional expenditure alongside the covered cancer therapy by the statutory or private health insurance in Germany. It is remarkable that interviewed patients not only reported actual costs but also anticipated financial disadvantages, e.g. prospective loss of income. This notion is further reinforced by the representatives of social services, who noted the increasing concern about the risk of financial bankruptcy due to a cancer diagnosis in recent years. To adapt these prospective financial disadvantages, the main category *objective financial* *disadvantages* was subdivided into *actual and anticipated financial* *disadvantages*.

### Coping strategies

The interviewed patients primarily revealed cognitive efforts to deal with financial disadvantages. Seven cognitive coping strategies could be established. They encompass mental strategies to regulate emotions and challenges with actual and anticipated financial changes. Behavioural coping strategies, such as “increasing financial resources” and “reducing financial spendings”, were mainly reported by the representatives of the social services and in previous studies. All coping strategies are listed in Table 3.

**Table 3:** Cognitive and behavioural strategies to cope with financial effects of cancer experienced by patients

Category	Subcategory	Source		
		Patient	Social services	Literature review
Cognitive coping strategies	Downward comparison with others	x		
	Trust in the health and welfare system	x		
	Acceptance of the financial situation and the disease	x	x	
	Taking things as they come	x		
	Being preoccupied with other problems (health before finances)	x		
	Repression of thoughts of the expected financial disadvantages	x		
	Information seeking	x	x	
Behavioural coping strategies	**Increase financial resources**			
	Applying for donation from non-governmental organisations or additional compensation payments from social- and welfare system		x	[29]
	Return-to-work early on		x	[32]
	Using savings			[27, 28, 30, 32, 35, 38, 39]
	Borrowing money or incurring debt		x	
	Accepting additional work		x	
	**Reduce financial spending**			
	Food	x	x	[28, 32]
	Housing	x	x	[28, 32]
	Leisure activities	x		[28, 32]

### Subjective financial distress

As a consequence of actual and anticipated financial disadvantages and coping strategies, cancer patients can experience subjective distress in different aspects of daily living. Table 4 summarises the impact on eight identified aspects and refers to the corresponding sources.

**Table 4:** Subjective financial distress experienced by cancer patients

Life area	Patients (P) and social services (S)	Literature review
Employment	The effect of cancer on work is multi-layered. Not only consequences of income losses like housholds’ ability to make ends meet **(S)** but also the missing professional self-fulfillment opportunities cause distress **(P)**. The latter becomes more relevant when (self-)employment must be given up and retirement starts earlier on **(P)**. Further distress arises regarding return-to-work options **(P; S)** Subjective financial distress also arises due to missing education that qualifies for (new) employment, especially for younger cancer survivors **(S)**	[32, 40–42]
Living situation	The actual and/or anticipated costs also determine decisions regarding the living situation. Although the wish to live in a single apartment is present, especially younger patients decide to stay in a shared flat with family members or friends. Life planning e.g. buying a house for the family, is deprioritized while property owners consider to terminate their loan payment and sell their homes **(P, S)**	[27, 31, 32, 37, 43]
Family	Familiar integration weakens as patients experience worries and distress about the financial situation of their family members due to their cancer disease. This situation could affect partners who might reduce their working hours to care for the patient or the children who may, for instance, still be in education **(S)**	[24–28, 30, 37, 38, 44]
Social participation	The loss of financial resources threatens patients’ ability to participate in society, such as leisure activities and vacation planning. Patients reduce their personal expenses for recreational activities to cope with the financial situation. This leads to changes in the individual standard of living and, therefore, affects the patients’ social identity. Maintaining social contact, e.g. going out for a meal or giving presents to loved ones, is difficult when patients are under financial pressure **(P)**	[28]
Health promoting lifestyle	Subjective financial distress can be experienced when a health-promoting lifestyle can not be achieved anymore due to financial issues, such as cancelling fitness centre subscriptions or buying cheap but unhealthy food **(P)**	
Additional personally preferred treatments	Cancer treatment can affect individual health considerations, e.g. fertility or utilisation of complementary medicine **(S, P)**. In Germany, not all legal assisted reproduction techniques and treatments or personally preferred complementary medicine are covered by health insurance. Thus, some patients feel distressed as they do not have the financial resources to pay it on their own	[24, 32]
Navigating health system	Patients must cooperate with health insurers and social security providers to obtain payments. These interactions are frustrating and exhausting **(P; S)**. Five categories of distress were identified: (1) extensive effort, (2) communication with providers, (3) insufficient knowledge and problem of comprehension about rights, benefits, responsibility and counselling, (4) compulsion to make decisions, and (5) delays of payment	[25, 28–30, 43]
Unspecific	Patients also reported unspecific and diffuse financial distress between or across different aspects of daily living **(P)**. Patients are often frustrated as plans of living change or they feel ashamed about (anticipated) financial issues. A greater level of existential anxiety among cancer patients can be observed in the last couple of years **(S)**	[24, 33, 45]

### Conceptual model to describe financial effects of cancer experienced by patients

**Fig. 1:**
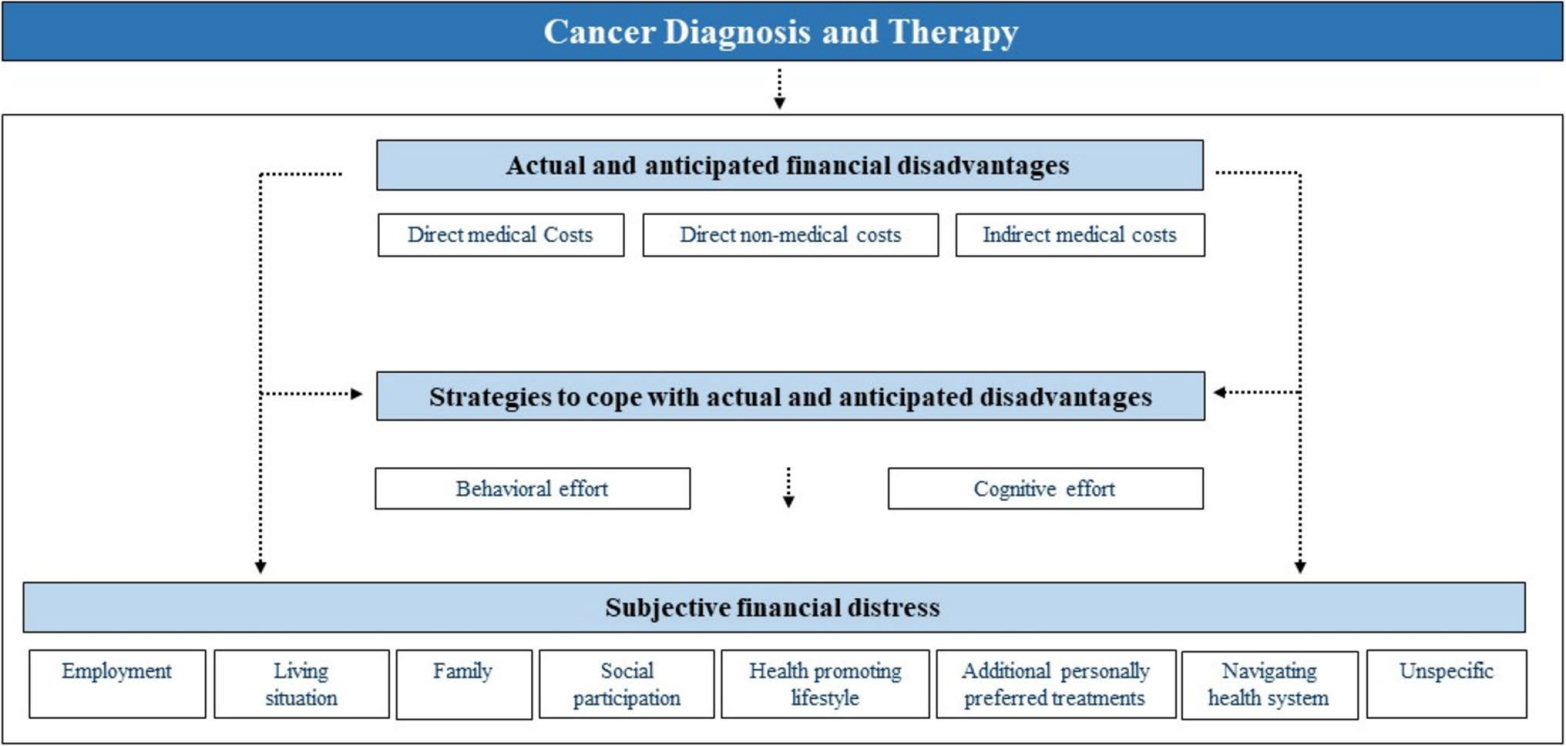
Conceptual model to describe financial effects of cancer experienced by patients

Based on the presented results, a conceptual model to describe the financial effects of cancer experienced by patients was developed (Fig. 1). The construct is named as “financial effects” to highlight the entire mechanism of the three proposed dimensions rather than focussing on the outcome as “financial toxicity”. Thus, all identified dimensions interact with one another while the degree of each subdimension can differ for individuals due to the personal perception of the financial effects experienced by their cancer diagnosis and therapy. Furthermore, the proposed model and its derived dimensions are driven by political, economic, and societal developments as well as individual risk factors.

#### Actual and anticipated financial disadvantages

We suggest clarifying the common term *objective financial burden* by subdividing it into *actual* and *anticipated*. Furthermore, we argue to rename the term *burden* into a more concrete expression, such as *disadvantages*. In line with previous literature and models of financial effects, we divided actual and anticipated disadvantages in *direct medical*, *direct non-medical*, and* indirect costs.*

#### Strategies to cope with actual and anticipated financial disadvantages

Actual and anticipated financial disadvantages can either directly culminate in subjective financial distress or via the maladaptive and dysfunctional use of coping strategies that an individual applies as a consequence of the perceived financial disadvantages.

#### Subjective financial distress

Subjective financial distress can be conceptualised as negative effects from actual or anticipated financial disadvantages on areas of daily living. These areas of daily living can be divided into employment, living situation, family, social participation, health-promoting lifestyle, additional personally preferred treatments, navigating the health system, and unspecific cross-category issues (Table 2). The differentiation of subjective financial distress regarding affected aspects of daily living allows a true-to-life understanding that is patient-oriented.

## Discussion

### Main finding of this study

This study represents a stakeholder-informed conceptualisation of financial effects in the course of a tumour disease for the German health- and welfare context based on our empirical results. Building on the conceptual model presented, we will develop the first German PROM to assess subjective financial distress.

Informed by a systematic literature review, qualitative interviews with cancer patients, and a focus group with social services representatives, we identified three construct dimensions for our model. The first dimension consists of actual and anticipated financial disadvantages, divided into (anticipated) direct medical/non-medical costs and indirect costs. These financial disadvantages could affect subjective financial distress directly or indirectly. In the latter, the impact on distress is moderated by coping strategies. This second dimension includes not only behavioural efforts to deal with actual and/or anticipated financial disadvantages, but also cognitive efforts. In the third dimension, subjective financial distress is operationalised and concretised in different aspects of daily living in which distress can be expressed.

It should be noted that data collection for this study took place during the COVID-19 pandemic, which may have influenced participants’ experiences and our findings. In particular, interviews with patients revealed that the pandemic led to reduced personal expenditures, potentially affecting perceptions of financial burden[19]. Questions related to the pandemic were explicitly included in both the patient interview guide and the focus group guide. In contrast, COVID-19 did not emerge as a relevant topic during the focus groups with social services representatives.

### What is already known on this topic

Some universal models describing financial distress in the context of a cancer diagnosis and treatment have already been published. One of the first was presented by Carrera et al. in 2018 [3]. In this model, subjective financial distress results from cancer-related expenditures and reduction in wealth and its combination with the patient’s anxiety and discomfort of their cancer experience. Witte et al. [5] proposed a model in which objective financial distress including direct and indirect costs leads to subjective financial stress, which consists of a material, psychosocial, and behavioural component. Subjective financial distress, in turn, can contribute to financial toxicity [5]. In the model by Lueckmann et al. [46], that is based on the model of Carrera et al., bureaucracy is integrated as one determent for subjective financial distress [46]. Furthermore, they found that the perception of varying levels of subjective financial distress depends on the financial adaptations made in response to objective costs. These coping strategies were categorised into a reduction in expenditure and an increase in resources 46. Lueckmann et al. [46] mentioned expected costs as a possible source of distress but do not include this, in our opinion, enormous relevant dimension in their model [46].

### What this study adds

Common to all three models is the distinction between objective and subjective financial distress in common, which we would like to build upon. However, we propose a model that is characterised by additional and specified dimensions. First, we systematically concretised the model by dividing objective financial distress into actual and anticipated disadvantages in terms of indirect costs, direct medical costs and direct non-medical costs. Thus, we underline the enormous impact of expectations and anticipation on subjective financial distress. It is remarkable that the subcategories of direct (non)- medical costs we established, are consistent with those of Lueckmann et al. [20] (Table 2). Second, our model focuses not only on behavioural effort, as pre-existing models do, but also on cognitive effort. Both cognitive and behavioural reactions mediate between stressors and distress, manage the source of stress, and regulate emotional responses [19, 47]. Although cognitive coping strategies have not been considered in models on financial hardship of cancer patients, our results are partially in line with the study by Head et al. [48] on coping with financial consequences of cancer (e.g. information seeking, acceptance or repression of thoughts and sorrows) [48]. Future research should investigate the extent to which cognitive coping strategies influence subjective financial distress and whether they should be included in PROMs designed to assess financial burden. While the prevalence and impact of these strategies remain to be empirically determined, it appears important to keep them in view, particularly in the context of patient counselling and support. Acknowledging that patients also employ cognitive strategies to manage financial distress is central to holistic care, as it enables healthcare providers to address not only material or behavioural needs but also patients’ internal coping efforts. Third, as in previous models subjective financial distress remains vague, we developed a structured and specified domain of subjective financial distress, which is positioned in daily life and every day practices, e.g. including job-related challenges, limited social participation and bureaucratic obstacles. This conceptualisation of subjective financial distress is an added value in particular for a structured social services counselling, as the specific areas in which support is needed can be determined intuitively.

To our knowledge, this is the first comprehensive model for the German context that provides the conceptual basis for the development of a PROM to capture financial effects of cancer experienced by patients and to assess their subjective financial distress in the future. Our analyses indicated that healthcare-specific characteristics need to be incorporated into the model as country-specific stressors mediate the distress. The German healthcare setting is characterised by a complex interplay of healthcare and welfare stakeholders. The compulsory health insurance, as either statutory (90% of the German population) or private, covers most healthcare costs, so that the extent of out-of-pocket costs is limited. During illness, sickness benefits are legally mandated for all individuals to mitigate income losses, providing approximately 70% of an individual’s regular gross income for up to 78 weeks upon application. Additionally, welfare benefits such as unemployment benefits or reduced earning capacity pensions can be claimed. These characteristics determine the degree of actual and, especially, anticipated objective financial burden. However, it is difficult to navigate the German system due to the various parties involved in applying for support benefits, which leads to distress caused by administrative hurdles. The influential component of country-specific characteristics also applies to other aspects of daily living. While coping strategies refer to the individual and occur universally, they can also be influenced by the socialisation of society. This country-specific approach is supported by another previously introduced model for the Italian context by Riva et al. [49] which is comparable with our conceptualisation in terms of complexity of categories and relevance to everyday life [49]. Thus, the categories family, job, and bureaucracy are in line with the categories we call family, employment, and navigating the health system. Comparing our model items with results from studies in other countries, it becomes evident that the categories, we identified, are universal, while the content of each category remains country-specific. Our model could therefore also be adjusted to other countries with universal healthcare coverage.

We propose to understand the model as the overall “financial effects of cancer experienced by patients” instead of describing it in terms of “financial toxicity”. The term “toxicity” reflects the extent to which something is harmful to the cancer patients, and we argue that it reflects the outcome rather than the multi-dimensional construct itself. However, it is necessary to measure the entire construct to determine patients already at risk for financial toxicity and to understand the phenomena exhaustively.

Based on the presented conceptual model, we will develop and validate a questionnaire as a new PROM for financial effects of cancer in Germany using a large sample of patients suffering from different types of cancer. The results of this quantitative study will shed light on the correlation of and interaction between the different domains in our proposed model [17].

### Limitations of this study

Despite careful selection of the study design, our study has limitations. One limitation might be the time gap between data collection and publication of the framework, which could potentially influence the interpretation of our identified mechanism for subjective financial distress in Germany. However, to our knowledge, no structural changes of the German healthcare setting have emerged in the interim that would alter our results. Further studies that have been published after our review were thoroughly investigated, but as far as we know, did not provide new evidence that was previously neglected in our presented framework [50]. It should be noted that the recruitment of patients who were at high risk of experiencing subjective financial distress was limited. The majority of the interviewed patients were married, employed, and received sick pay. Consequently, the perspectives of elderly individuals or cancer survivors may have been underestimated in our findings. Lastly, due to COVID-19 restrictions, the interviews in the outpatient setting had to be conducted using online videoconferencing software. Some interviews experienced minimal technical issues that may have influenced the flow of the narratives. The same can be applied to the focus group participants of the social services. Moreover, the systematic literature review focussed on subjective financial distress, as we assumed that this concept had not been studied exhaustively. This assumption might have led to an underestimation of other dimensions in our introduced model, even though we addressed them in our qualitative studies. Furthermore, although the systematic literature review identified a total of 8 out of 46 studies from Germany, the majority of studies were carried out in other healthcare settings. To increase comparability, we only included studies from high-income countries with universal healthcare coverage and matched results with identified dimensions of our qualitative studies using a qualitative content analysis.

## Conclusions

Financial effects of cancer experienced by patients is a multidimensional construct. We propose a conceptual model that comprises actual and anticipated objective financial disadvantages, strategies to cope with them in terms of cognitive and behavioural efforts as well as subjective financial distress in different aspects of daily living. While the identified dimensions can be observed in countries with universal healthcare coverage, the content and degree of each category depend on country-specific characteristics. The interaction of systemic factors and personal coping mechanisms determines the individual perception of the financial effects of cancer experienced by patients. This requires a country-specific approach to analyse and understand the phenomenon. Based on the proposed conceptual model, the development of a new PROM for financial effects of cancer in the German healthcare context is needed to explore the interaction of dimensions and to assess the degree of subjective financial distress in patients.

## Data Availability

No datasets were generated or analysed during the current study.
